# Supplementary material for: Analysis of PPARγ Signaling Activity in Psoriasis
Source: Int J Mol Sci. 2021 Aug 10;22(16):8603. doi: 10.3390/ijms22168603 (PMC8395241; doi:10.3390/ijms22168603)
Supplement: Supplementary file 1 [file ijms-22-08603-s001.zip › Supplemental materials_Analysis of PPARg signaling activity in psoriasis/Pathway models/Models images and html files/PPARG expressed in cells assotiated with psoriasis/115840.html]

adipose tissue


# Tissue adipose tissue

|  |  |
| --- | --- |
| URN | urn:agi-ncimtissue:C0001527 |
| Total Entities | 16 |
| Connectivity | 7597 |
| Name | adipose tissue |

---

|  |  |
| --- | --- |
| ChildConcepts | abdominal fat |
|  | subcutaneous fat |
|  | mammary gland fat |
|  | white adipose tissue |
|  | gonadal adipose tissue |
|  | pericardial fat |
|  | beige adipose tissue |
|  | epicardial fat |
|  | perivascular adipose tissue |
|  | fat body |
|  | brown adipose tissue |
|  | inguinal fat |
|  | interscapular fat |
|  | mesenteric fat |
|  | perirenal fat |
|  | epididymis fat |

---

|  |  |
| --- | --- |
| Pathway | PPARG expressed in cells assotiated with psoriasis |

---

|  |  |
| --- | --- |
| MedScan ID | 6007680 |

---

|  |  |
| --- | --- |
| Alias | fat tissues |
|  | fatty tissue |
|  | fat tissue |
|  | yellow adipose tissue |
|  | yellow adipose tissues |
|  | adipose tissue |
|  | adipose tissues |
|  | fatty tissues |

---

|  |  |
| --- | --- |
| NCIm ID | C0001527 |

---
